# Влияние пульс-терапии метилпреднизолоном на показатели углеводного обмена у детей с хроническими аутоиммунными и аутовоспалительными заболеваниями

**DOI:** 10.14341/probl13606

**Published:** 2026-09-08

**Authors:** А. В. Витебская, В. А. Подзолкова

**Affiliations:** Первый Московский государственный медицинский университет им. И.М. Сеченова (Сеченовский Университет) Россия; I.M. Sechenov First Moscow State Medical University (Sechenov University) Russian Federation

**Keywords:** пульс-терапия, глюкокортикоиды, глюкоза крови, катамнез, pulse therapy, glucocorticoids, blood glucose, follow-up

## Abstract

**ОБОСНОВАНИЕ:**

ОБОСНОВАНИЕ. Применение высоких доз глюкокортикостероидов (ГКС) может приводить к развитию гипергликемии. Пульс-терапия (ПТ) ГКС обладает меньшими побочными эффектами по сравнению с длительным пероральным приемом ГКС у взрослых пациентов. Контроль гликемии на фоне ПТ ГКС в детском возрасте малоизучен.

**ЦЕЛЬ:**

ЦЕЛЬ. Изучить краткосрочные и долгосрочные эффекты ПТ метилпреднизолоном на показатели углеводного обмена детей с хроническими аутоиммунными и аутовоспалительными заболеваниями

**МАТЕРИАЛЫ И МЕТОДЫ:**

МАТЕРИАЛЫ И МЕТОДЫ. Проанализированы показатели углеводного обмена 15 пациентов (13 девочек, 2 мальчика) до, на фоне и после ПТ метилпреднизолоном (3 введения; 0 ч, 24 ч, 48 ч; суммарная доза 27,0 (23,0; 31,6) мг/кг). Наблюдение продолжалось от 0,2 до 6,3 лет.

**РЕЗУЛЬТАТЫ:**

РЕЗУЛЬТАТЫ. Во время ПТ уровень гликемии превышал нормальные значения у всех пациентов через 4-10 ч после начала ПТ, достигая пика через 7 ч – 10,8 (9,9; 11,8) ммоль/л. Пики гликемии через 7 ч после второго и третьего введения были статистически значимо ниже по сравнению с первым днем – 9,1 (8,2; 10,7) ммоль/л (p<0,05) и 8,9 (7,4; 9,2) ммоль/л (p<0,01) соответственно. Через 24 ч после каждого введения ГКС отмечалась тенденция к нормализации гликемии. Значения гликемии через 72 ч не коррелировали с возрастом, SDS ИМТ, дозой метилпреднизолона и пиковыми значениями гликемии в ходе ПТ. Статистически значимых различий между показателями гликемии пациентов, впервые перенесших ПТ, и пациентов, имевших в прошлом опыт ПТ, выявлено не было.

Наблюдение в течение 2,7 (0,5; 4,6) лет не выявило отсроченных осложнений.

**ЗАКЛЮЧЕНИЕ:**

ЗАКЛЮЧЕНИЕ. ПТ ГКС у детей приводила к краткосрочной ГКС-индуцированной гипергликемии. Максимальные значения гликемии отмечались через 7 ч после каждого введения ГКС; после повторных введений ГКС подъем гликемии был менее выражен, чем после первого введения. Спонтанная нормализация гликемии отмечалась через 24 часа после введения метилпреднизолона. Мы не выявили каких-либо отсроченных нарушений углеводного обмена.

## Abstract (en)

**BACKGROUND:**

BACKGROUND: The use of glucocorticosteroids (GCS) in high doses can lead to hyperglycemia. GCS pulse therapy (PT) cause fewer side effects compared to long-term oral administration of GCS in adult patients. Glycemic control in children while GCS PT is not well studied.

**AIM:**

AIM: To study short- and long-term effects of methylprednisolone pulse therapy on carbohydrate metabolism parameters in children with autoimmune and autoinflammatory diseases

**MATERIALS AND METHODS:**

MATERIALS AND METHODS: We analyzed carbohydrate metabolism parameters of patients before, during, and after PT with methylprednisolone (3 infusions; 0 h, 24 h, 48 h; total dose 27.0 (23.0; 31.6) mg/kg). Observation lasted for 0.2–6.3 years.

**RESULTS:**

RESULTS: We analyzed data from 15 patients (13 girls, 2 boys). During PT, the glycemia level exceeded normal rages in all patients in 4-10 hours after the start of PT, reaching a peak in 7 h – 10.8 (9.9; 11.8) mmol/l. Glycemia peaks 7 h after the second and the third infusions were significantly lower compared to the first day – 9.1 (8.2; 10.7) mmol/l (p<0.05) and 8.9 (7.4; 9.2) mmol/l (p<0.01), correspondently. A tendency toward normalization of glycemia was noted in 24 h after each administration of GCS. Glycemic values in 72 h did not correlate with age, SDS BMI, methylprednisolone dose, and peak glycemic values during PT. There were no significant differences between the glycemic indices of patients undergoing PT for the first time and those who already had experience of PT. Observation for 2.7 (0.5; 4.6) years did not reveal any delayed complications.

**CONCLUSION:**

CONCLUSION: GCS PT in children resulted in short-term GCS-induced hyperglycemia. Peak glycemia values were observed in 7 hours after each GCS infusion; the rises of glycemia after repeated infusions were lower than after the first one. Spontaneous glycemia normalization was observed in 24 hours after methylprednisolone administration. We did not reveal any delayed disorders of carbohydrate metabolism.

## ОБОСНОВАНИЕ

Препараты глюкокортикостероидов (ГКС) — это обширная группа синтетических гормонов коры надпочечников, применяющихся с противовоспалительной целью у пациентов с аутовоспалительными и аутоиммунными заболеваниями. Несмотря на высокую эффективность, использование ГКС ограничено широким спектром побочных эффектов. Назначение ГКС приводит к ятрогенному гиперкортицизму, для которого типичны осложнения, описанные при эндогенном избытке кортизола, в том числе нарушения углеводного обмена [1][2].

Пульс-терапия (ПТ) ГКС является предпочтительным методом лечения при состояниях, требующих быстрого иммунодепрессивного и противовоспалительного эффектов. Гипергликемия — частый побочный эффект ПТ. Исследования, посвященные изучению краткосрочных и долгосрочных осложнений ПТ, показали хорошую переносимость ПТ по сравнению с длительным приемом ГКС внутрь. Однако в большинстве исследований принимали участие взрослые пациенты, а влияние ПТ на контроль гликемии в детском возрасте малоизучено [3][4][5].

## ЦЕЛЬ ИССЛЕДОВАНИЯ

Изучить краткосрочные и долгосрочные эффекты ПТ метилпреднизолоном на показатели углеводного обмена у детей с хроническими аутоиммунными и аутовоспалительными заболеваниями.

## МАТЕРИАЛЫ И МЕТОДЫ

## Место и время проведения исследования

Место проведения. Университетская детская клиническая больница (в настоящее время — Сеченовский центр материнства и детства) ФГАОУ ВО Первый МГМУ им. И.М. Сеченова Минздрава России.

Время исследования. Проанализированы медицинские карты пациентов, которым в период с сентября 2018 по май 2019 гг. проводился контроль гликемии на фоне ПТ метилпреднизолоном. Повторные обследования пациентов были выполнены в период с апреля 2019 по июнь 2024 гг.

## Изучаемые популяции

Популяция. В исследование были включены пациенты с хроническими аутоиммунными и аутовоспалительными заболеваниями без нарушений углеводного обмена.

Критерии включения: 1) девочки и мальчики младше 18 лет; 2) ПТ метилпреднизолоном в течение 3 суток в суммарной дозе 750–2500 мг; 3) наличие результатов исследования гликемии с помощью глюкометра на фоне ПТ (не менее четырех измерений в сутки).

Критерии исключения: наличие нарушений углеводного обмена по данным анамнеза (например, сахарного диабета 1 типа) и результатам исследования глюкозы плазмы натощак (ГПН) до проведения ПТ.

## Способ формирования выборки из изучаемой популяции (или нескольких выборок из нескольких изучаемых популяций)

путем сплошного включения наблюдений.

## Дизайн исследования:

одноцентровое, интервенционное, динамическое, ретроспективное, частично проспективное (пациенты могли наблюдаться в клинике до достижения 18 лет; длительность наблюдения составила от 0,5 до 6,3 года).

## Описание медицинского вмешательства (для интервенционных исследований)

По назначению врача-ревматолога пациентам проводилась ПТ: в течение трех суток 1 раз в день внутривенно капельно вводился метилпреднизолон с 0,9%-ным раствором натрия хлорида; повторные инфузии проводились через 24 и 48 ч. До проведения ПТ всем пациентам проводилось исследование ГПН и гликированного гемоглобина (HbA1c).

В период проведения ПТ метилпреднизолоном пациентам предлагалось дополнительно измерять гликемию. После подписания информированного согласия на медицинское вмешательство гликемия измерялась с помощью индивидуальных глюкометров через 2, 4, 7, 10, 21, 24, 28, 31, 34, 45, 48, 52, 55, 58, 69, 72 ч после первого введения препарата.

Пациенты получали питание в соответствии с общепринятым в стационарах столом ОВД (основной вариант стандартной диеты); на время проведения ПТ рекомендовалось исключить дополнительное питание, сладкие продукты и напитки, в том числе соки.

## Методы

На основании данных о росте и массе тела рассчитывался индекс массы тела (ИМТ), в соответствии с нормативами Всемирной организации здравоохранения вычислялся SDS ИМТ, на основании чего делалось заключение о дефиците массы тела (ниже -2,0), нормальной массе тела (от -2,0, до +1,0), избытке массы тела (выше +1,0, до +2,0), ожирении 1 степени (выше +2,0, до +2,5) или 2-й степени (выше +2,5, до +3,0).

Оценивались показатели углеводного обмена: ГПН (в норме <6,1 ммоль/л), HbA1c (в норме <6,0%). При наличии ожирения пациенту проводился пероральный глюкозотолерантный тест (ПГТТ) с определением ГПН (в норме <6,1 ммоль/л) и глюкозы плазмы через 2 ч (в норме <7,8 ммоль/л) после стандартной нагрузки глюкозой (1,75 г/кг, но не более 75 г).

В период проведения ПТ пациенты измеряли гликемию с помощью глюкометров Контур ТС, соответствующих ГОСТ Р ИСО-15197-2015.

## Статистический анализ

Статистический анализ проводился с использованием программы StatTech v. 4.8.1 (разработчик — ООО «Статтех», Россия). Количественные показатели оценивались на предмет соответствия нормальному распределению с помощью критерия Шапиро-Уилка. Поскольку распределение большинства изученных признаков было отличным от нормального, применяли методы непараметрической статистики — данные описывались с помощью медианы (Me) и нижнего и верхнего квартилей (Q1; Q3). Для сравнения двух связанных совокупностей использовали критерий Вилкоксона, для выявления корреляций — коэффициент Спирмена. Минимальную вероятность справедливости нулевой гипотезы принимали при 5% уровне значимости (р<0,05).

## Этическая экспертиза

Работа выполнена в рамках научно-исследовательской работы «Медикаментозный синдром Иценко-Кушинга у детей», протокол одобрен Локальным этическим комитетом ФГАОУ ВО Первый МГМУ им. И.М. Сеченова Минздрава России 18.05.2023 (протокол №09-23).

## РЕЗУЛЬТАТЫ

Проанализированы данные 15 пациентов (13 девочек, 2 мальчика). ПТ ГКС была назначена с целью лечения ювенильного идиопатического артрита (7 пациентов); ювенильной системной склеродермии (5 пациентов); панникулита Вебера-Крисчена, язвенного колита, неспецифического аортоартериита Такаясу (по 1 пациенту). Пять пациентов из 15 имели опыт ПТ в прошлом, пероральные препараты ГКС на момент проведения ПТ не назначались. До ПТ у всех пациентов показатели углеводного обмена соответствовали нормальным значениям. Суммарная доза метилпреднизолона при проведении ПТ составила 1495 (1375; 1500) мг (27,0 (23,0; 31,6) мг/кг). Характеристики пациентов представлены в табл. 1.

**Table:** Таблица 1. Характеристики пациентов при первичном обследовании и при последнем наблюдении Примечание: ¹ p>0,05 — статистически значимых различий с аналогичным показателем при первичном обследовании не выявлено.

Характеристика	При первичном обследовании	При последнем наблюдении
Возраст	14,9 (11,5; 16,4) года	17,6 (17,3; 17,8) года
SDS ИМТ	+0,59 (-0,52; +1,19)Из них:9 — нормальные значения;3 — избыток массы тела (SDS ИМТ +1,12, +1,25, +1,36);1 — дефицит массы тела (SDS ИМТ -2,87);1 — ожирение 1-й (SDS ИМТ +2,07);1 — ожирение 2-й степени (SDS ИМТ +2,99)	+0,70 (-0,40; +0,98)¹Из них:12 — нормальные значения;2 — избыток массы тела (SDS ИМТ +1,92 и +1,98);1 — ожирение 2-й степени (SDS ИМТ +2,75)
Гликированный гемоглобин HbA1c	5,1 (5,0; 5,7) %	-
Глюкоза плазмы натощак	4,7 (4,4; 5,0) ммоль/л	4,3 (4,0; 4,5) ммоль/л¹

В период проведения ПТ уровень гликемии превышал нормальные значения у всех пациентов через 4–10 ч после начала ПТ, достигая пика через 7 ч — 10,8 (9,9; 11,8) ммоль/л. Аналогичные пики гликемии через 7 часов после второго (31 ч) и третьего (55 ч) введения были статистически значимо ниже по сравнению с первым днем — 9,1 (8,2; 10,7) ммоль/л (p<0,05) и 8,9 (7,4; 9,2) ммоль/л (p<0,01) соответственно (рис. 1, табл. 2).

**Figure:**
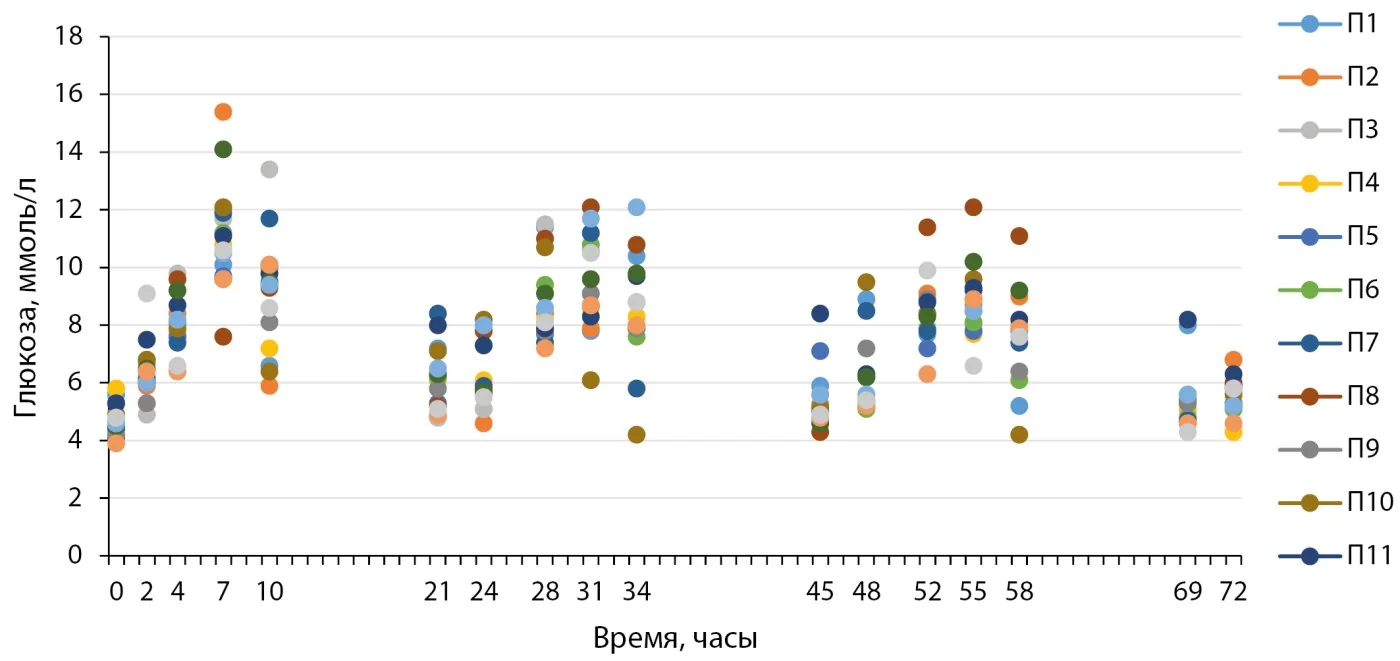
Рисунок 1. Показатели гликемии в период проведения пульс-терапии метилпреднизолоном.

**Table:** Таблица 2. Показатели гликемии по данным глюкометра на фоне пульс-терапии Примечание: ¹ p<0,05, ² p<0,01 — статистически значимые различия с аналогичным показателем в день 1.

Время с момента введения метилпреднизолона	Показатели гликемии, ммоль/л
День 1	День 2	День 3
2 ч; 26 ч; 50 ч	6,2 (6,0, 6,8)	-	-
3 ч; 27 ч; 51 ч	-	8,4 (7,9; 10,1)	8,4 (7,8; 8,9)
4 ч; 28 ч; 52 ч	8,1 (7,7; 8,6)	-	-
7 ч; 31 ч; 55 ч	10,8 (9,9; 11,8)	9,1 (8,5; 10,7)¹	8,7 (8,1; 9,3)²
10 ч; 34 ч; 58 ч	9,4 (7,7; 10,1)	8,3 (7,9; 9,8)	7,8 (6,4; 8,2)
21 ч; 45 ч; 69 ч	6,1 (5,2; 6,8)	5,1 (4,9; 5,6)	5,1 (4,7; 5,5)
24 ч; 48 ч; 72 ч	5,8 (5,6; 6,7)	5,6 (5,3; 6,8)	5,6 (5,2; 5,9)
Площадь под кривой гликемии, ммоль/л * час	194,4 (176,9; 213,0)	182,4 (177,7; 205,1)	168,5 (166,0; 184,1)²

Через сутки после каждого введения ГКС (24, 48 и 72 ч) отмечалась тенденция к нормализации гликемии (рис. 1). Площадь под кривой (ППК) гликемии после каждого следующего введения метилпреднизолона была меньше, чем в предыдущий день; статистически значимые различия (p<0,01) получены при сравнении ППК в первый и третий дни (табл. 2).

Через 72 ч после первого введения метилпреднизолона гипергликемия натощак сохранялась лишь у двух пациентов — девушки 15,9 года и юноши 17,5 года (пациенты №2 и №11; 6,8 и 6,3 ммоль/л соответственно); гликемия самостоятельно нормализовалась через сутки. Значения гликемии через 72 ч не коррелировали с возрастом, SDS ИМТ, дозой метилпреднизолона и пиковыми значениями гликемии в ходе ПТ.

Статистически значимых различий между показателями гликемии пациентов, впервые перенесших ПТ, и пациентов, имевших в прошлом опыт ПТ, выявлено не было.

При контрольном обследовании через 6 мес пациентов, сохранявших гипергликемию через 72 ч от начала ПТ (№2 и №11), показатели ГПН были в пределах нормальных значений (4,30 и 3,58 ммоль/л); исследование HbA1c удалось провести только у пациента №11 (5,5%), так как у пациентки №2 на момент обследования была выявлена анемия (гемоглобин 77–84 г/л), ассоциированная с основным заболеванием. При повторном обследовании через 1 год — HbA1c — 5,1%.

Двум пациентам с ожирением после завершения ПТ был проведен ПГТТ. Показатели гликемии на фоне ПТ юноши с ожирением 1 степени (пациент №12) не отличались от показателей других пациентов. ПГТТ не выявил нарушений углеводного обмена; отмечена умеренная инсулинорезистентность. В дальнейшем ГКС не назначались. При последнем наблюдении в 17,8 года у пациента была нормальная масса тела, нарушения углеводного обмена отсутствовали (табл. 3).

**Table:** Таблица 3. Характеристики и результаты обследования пациентов с ожирением Примечание: ИМТ — индекс массы тела, HbA1c — гликированный гемоглобин, индексы HOMA, Caro — индексы инсулинорезистентности.

Пол	Возраст, стадия полового созревания, рост, вес (SDS ИМТ) при проведении ОГТТ	HbA1c	Инсулин, индексы инсулино-резистен-тности	Результаты перорального глюкозотолерантного теста	Возраст, стадия полового созревания, рост, вес (SDS ИМТ), показатели ГПН при последнем наблюдении
Исходно	Через 2 часа
Пациентка №8	11,3 года, Таннер 3, 154 см, 71,5 кг (SDS ИМТ +2,99)	5,5%	22,5 Ед/л; HOMА 4,5; Сaro 0,2	4,5 ммоль/л	5,8 ммоль/л	17,6 года, Таннер 5, 169 см, 93,4 кг (SDS ИМТ +2,75); ГПН 4,3 ммоль/л
12,2 года, Таннер 3, 157 см, 73,0 кг (SDS ИМТ +2,67)	5,0%	17,2 Ед/л; HOMА 4,5; Сaro 0,2	3,8 ммоль/л	4,4 ммоль/л
Пациент №12	12,2 года, Таннер 2, (SDS ИМТ 2,04)	5,9%	23,0 Ед/л; HOMА 4,4; Сaro 0,2	4,3 ммоль/л	7,6 ммоль/л	17,8 года, Таннер 5, 180 см, 75 кг (SDS ИМТ +0,81); ГПН 4,7 ммоль/л

Девушка с ожирением 2-й степени (пациентка №8) была единственной, у кого на 3-и сутки ПТ выявлялись значения гликемии, соответствующие критериям СД (11,4–12,1–11,1 ммоль/л через 52–55–58 ч). ПГТТ не выявил нарушений углеводного обмена (табл. 3). В дальнейшем пациентке был назначен пероральный прием метилпреднизолона (начальная доза 25 мг (0,35 мг/кг) в пересчете на преднизолон) с последующим постепенным снижением дозы. На этом фоне показатели ГПН, определяемые амбулаторно каждые 3 месяца, оставались в пределах нормативных значений. Повторное обследование проведено через 11 мес на фоне сохраняющегося ожирения, продолжающегося приема метилпреднизолона (10 мг (0,15 мг/кг) по преднизолону). Нарушения углеводного обмена вновь были исключены (табл. 3).

Пероральный прием ГКС был полностью прекращен пациенткой №8 в 12,5 года. Показатели ГПН исследовались каждые 6 мес, всегда оставались в пределах нормальных значений. В связи с рецидивом основного заболевания повторные курсы ПТ ГКС по прежней схеме проводились в 14,9 и 16,5 года. На фоне ПТ отмечалось умеренное повышение гликемии (по данным самоконтроля) максимально — до 9,6 ммоль/л, с нормализацией гликемии на следующие сутки после окончания ПТ. После 16,5 года к терапии подключен тофацитиниб, терапия ГКС не возобновлялась. При плановом обследовании в 17,5 года, несмотря на сохраняющееся ожирение, уровень ГПН оставался в пределах нормальных значений (табл. 3).

Наблюдение за всей группой пациентов велось 2,7 (0,5; 4,6) года после проведения ПТ. За период наблюдения 3 пациента перенесли повторные курсы ПТ; 8 принимали ГКС в таблетированном виде; на момент последнего наблюдения пероральная терапия ГКС продолжалась у 4 пациентов в поддерживающих дозах 5–10 мг. Ни у кого не выявлено нарушений углеводного обмена. Характеристики пациентов на момент последнего наблюдения представлены в таблице 1.

## ОБСУЖДЕНИЕ

## Репрезентативность выборок

В выборке пациентов значительного преобладали лица женского пола, что обусловлено относительно высокой распространенностью хронических аутоиммунных и аутовоспалительных заболеваний среди девушек-подростков по сравнению с юношами [1][3]. В исследование были включены пациенты с различной массой тела — ИМТ варьировал от дефицита массы тела до ожирения 2-й степени. Набор участников исследования после ПТ проводился только в условиях федерального стационара, а наблюдение осуществлялось до достижения пациентами 18 лет, что могло способствовать «ускользанию» пациентов из-под наблюдения.

## Сопоставление с другими публикациями

В последние десятилетия терапевтические подходы к терапии аутоиммунных и аутовоспалительных заболеваний претерпели существенные изменения в связи с появлением и внедрением в клиническую практику новых препаратов. В то же время все больше внимания стали уделять безопасности применения ГКС и их влиянию на качество жизни пациентов [6][7].

Согласно данным литературы, несмотря на то, что гипергликемия отмечается даже при однократном введении ГКС [5], при ПТ она обычно протекает бессимптомно, не является предметом жалоб пациентов и не требует назначения дополнительной терапии [3].

На риск развития гипергликемии, индуцированной ГКС, могут влиять многочисленные факторы. В первую очередь, это выбор препарата и доза, путь и режим введения ГКС. Также повышают риски старший возраст и большее значение ИМТ пациента, наличие абдоминального ожирения и гипертриглицеридемии, семейный анамнез СД, нарушение функции почек, артериальная гипертензия, цитомегаловирусная инфекция, курение, раса и этническая принадлежность (например, афроамериканцы имеют повышенный риск), сопутствующий прием иммунодепрессантов или диуретиков [8].

ПТ оказывает существенно меньший долгосрочный эффект на гликемический контроль пациентов без предшествующих нарушений углеводного обмена, что является безусловным преимуществом. Например, после завершения терапии метилпреднизолоном у взрослых пациентов с аутоиммунным гепатитом нарушений углеводного обмена в группе ПТ не было выявлено, в отличие от групп пациентов, получавших ГКС перорально (27%) или комбинированные схемы пероральных препаратов и ПТ (26%) [4].

Наблюдательные исследования колебаний гликемии на фоне ПТ и приема высоких пероральных доз ГКС немногочисленны и посвящены разным препаратам и режимам введения для лечения различных нозологий. Результаты этих работ объединяет то, что у пациентов без предшествующего СД гипергликемия обычно развивается через несколько часов после введения препарата, сохраняется днем, вечером и ночью, но к следующему утру гликемия нормализуется [9][10][11]. В качестве примера можно привести одно из исследований с режимом ПТ, аналогичным нашему. При внутривенном введении 500 мг метилпреднизолона в течение трех дней отмечалась стойкая гипергликемия выше 11,1 ммоль/л примерно через 3 ч после начала инфузии, сохранявшаяся в течение дня, а самостоятельная нормализация гликемии зафиксирована к следующему утру [10]. Наши данные согласуются с опубликованными ранее — мы отмечали пик гликемии через 4–10 часов от введения метилпреднизолона с последующим самостоятельным снижением.

Существенным ограничением, затрудняющим проведения сравнения результатов нашего исследования с литературными данными, является то, что все приводимые работы выполнены с участием взрослых, а не детей. Нам удалось найти лишь одно рандомизированное плацебо-контролируемое исследование, в котором изучалась эффективность и безопасность пульс-терапии дексаметазоном при ювенильном идиопатическом артрите. У 2 пациентов из 30 отмечалась транзиторная гипергликемия выше 140 мг/л на фоне инфузии ГКС, но какие-либо подробности отсутствуют. Остается неясным, действительно ли у остальных пациентов не было подъема гликемии, или в момент наиболее вероятного пика просто не проводились измерения [11]. Стоит также обратить внимание на особенность одного исследования с относительно молодыми пациентами (средний возраст 32 года), где после дневных гипергликемий, вызванных пероральным приемом 1–2 мг/кг метилпреднизолона, на следующее утро у трети пациентов отмечались гипогликемии, что косвенно свидетельствует об отсутствии у них постоянной инсулинорезистентности [13].

Выраженность пика и длительность гипергликемии в различных исследованиях коррелируют с показателями ИМТ и гликированного гемоглобина [5][13][14]. Мы не выявили такой зависимости, однако длительная гипергликемия на 3-и сутки ПТ отмечалась только у пациентки с ожирением 2-й степени.

Полученные нами данные о тенденции уменьшения гипергликемического ответа на повторные введения ГКС в рамках ПТ согласуются с литературными данными. Например, в исследовании с участием женщин, получавших в течение 5 дней по 30 мг дексаметазона в рамках химиотерапии онкогинекологических заболеваний, гипергликемия была максимальна в первый день терапии, отмечалась после всех введений препарата, но на 5-й день значения гликемии были ниже, чем после первого введения ГКС [14]. Интересно, что подобная динамика гипергликемии согласуется с нашими данными полученными при наблюдении за детьми с сахарным диабетом 1 типа, которым в связи с тяжелыми системными заболеваниями назначалась ПТ ГКС — повышенная потребность в инсулине отмечались через 3–6 ч и сохранялись от нескольких часов до 3 суток после каждого введения [15].

Мы не выявили связь максимального уровня гликемии с предшествующей ПТ. Мнения исследователей по этому вопросу неоднозначны. В исследовании ПТ дексаметазоном в течение 4-х суток такой закономерности отмечено не было [9]. Еще в одной работе было зафиксировано, что гипергликемическая реакция у ГКС-«наивных» пациентов была ниже, чем у тех, кто до этого принимал аналогичные препараты в небольшой дозе [13]. С помощью систем непрерывного мониторинга глюкозы в период пандемии COVID-19 было продемонстрировано, что прием метилпреднизолона вызывал значительно больший гипергликемический эффект у пациентов без диабета, чем с ранее выявленным диабетом [16].

Ни одному из пациентов в рамках нашего исследования не потребовалась сахароснижающая терапия. Однако для поддержания нормогликемии у взрослых пациентов нередко используются различные режимы инсулинотерапии [1][17], а также опубликованы данные о попытках применения таблетированных препаратов, в частности митиглинида и репаглинида [1][10].

## Клиническая значимость результатов

Результаты нашего исследования подтвердили безопасность ПТ для пациентов детского возраста, продемонстрировав кратковременные гипергликемические реакции длительностью несколько часов после введения метилпреднизолона, не требовавшие назначения дополнительной терапии. Продемонстрирован катамнез пациентов, которые изначально могли быть отнесены к группе риска по развитию нарушений углеводного обмена (пациенты с ожирением и пациенты с гипергликемией через сутки после ПТ).

## Ограничения исследования

Основным ограничением исследования является малый объем выборки, что обусловлено необходимостью включить в исследование пациентов, получивших курс ПТ по одинаковому протоколу (для ПТ в реальной клинической практике применяются различные синтетические ГКС в различных дозах и режимах введения), а также связано с инвазивностью гликемического контроля с помощью глюкометра. Также результаты исследования могут быть искажены вследствие значительного преобладания в выборке лиц женского пола. Исследование HbA1c в катамнезе было ограничено отсутствием официальных рекомендаций по рутинному определению данного показателя у пациентов с аутоиммунными и аутовоспалительными заболеваниями, а также в связи с изменением общего анализа крови, ассоциированным с основным заболеванием.

## Направления дальнейших исследований

С учетом полученных результатов и литературных данных дальнейшие исследования планируются в направлении оценки того, какой вклад в развитие нарушений углеводного обмена вносит образ жизни пациентов, получающих ГКС для лечения хронических аутоиммунных и аутовоспалительных заболеваний.

## ЗАКЛЮЧЕНИЕ

ПТ ГКС у детей приводила к краткосрочной ГКС-индуцированной гипергликемии. Максимальные значения гликемии отмечались через 7 ч после каждого введения ГКС; после повторных введений ГКС подъем гликемии был менее выражен, чем после первого введения. Спонтанная нормализация гликемии отмечалась через 24 часа после введения метилпреднизолона.

По результатам наблюдения пациентов мы не выявили каких-либо отсроченных нарушений углеводного обмена.

## ДОПОЛНИТЕЛЬНАЯ ИНФОРМАЦИЯ

Источники финансирования. Работа выполнена по инициативе авторов без привлечения финансирования.

Конфликт интересов. Авторы декларируют отсутствие явных и потенциальных конфликтов интересов, связанных с содержанием настоящей статьи.

Участие авторов. Витебская А.В. — концепция и дизайн исследования, получение катамнестических данных, анализ результатов, написание статьи; Подзолкова В.А. — существенный вклад в дизайн исследования, получение и анализ данных пульс-терапии, внесение в рукопись существенной правки с целью повышения научной ценности статьи. Все авторы одобрили финальную версию статьи перед публикацией, выразили согласие нести ответственность за все аспекты работы, подразумевающую надлежащее изучение и решение вопросов, связанных с точностью или добросовестностью любой части работы.

Благодарности. Авторы выражают свою благодарность компании Ascensia Diabetes Care за бесплатно предоставленные для пациентов глюкометры и тест-полоски.
